# One novel *ACOT7–NPHP4* fusion gene identified in one patient with acute lymphoblastic leukemia: a case report

**DOI:** 10.1186/s12920-022-01378-7

**Published:** 2022-10-31

**Authors:** Xin Zong, Zhijie Kang, Dan Huang, Xuehong Zhang, Yuan Gao, Haina Wang, Weiling Li, Jinsong Yan

**Affiliations:** 1 Department of Hematology, Dalian Key Laboratory of Hematology, Liaoning Key Laboratory of Hematopoietic Stem Cell Transplantation and Translational Medicine, Liaoning Medical Center for Hematopoietic Stem Cell Transplantation, Dalian, 116044 China; 2 Diamond Bay Institute of Hematology, Second Hospital of Dalian Medical University, Dalian, 116027 China; 3 Department of Basic Medicine Academy, Dalian Medical University, Dalian, 116044 Liaoning China; 4 Institute of Cancer Stem Cell, Dalian Medical University, Dalian, China; 5 Department of Hematology, Second Hospital of Dalian Medical University, No. 467, Zhongshan Road, ShaHeKou District, Dalian, 116027 China

**Keywords:** Case report, Acute lymphoblastic leukemia, *ACOT7–NPHP4*, Fusion gene, Molecular biomarker

## Abstract

**Background:**

Acute lymphoblastic leukemia (ALL) is a type of heterogeneous hematopoietic malignancy that accounts for approximately 20% of adult ALL. Although ALL complete remission (CR) rate has increased to 85–90% after induction chemotherapy, 40–50% of patients eventually relapsed. Therefore, it is necessary to improve the outcomes of ALL via accurate diagnosis and individualized treatments, which benefits in part from molecular biomarkers. Here, we identified a new fusion gene, Acyl-CoA Thioesterase 7–Nephrocystin 4 (*ACOT7–NPHP4)*, in a 34-year-old patient with ALL. The fusion gene contributed to chemoresistance to doxorubicin and acted as a new molecular marker.

**Case presentation:**

A 34-year-old male patient was diagnosed with ALL (common B cell) based on clinical manifestations and laboratory results. Although the patient received two cycles of the hyper-CVAD-L regimen as chemotherapy, the induction treatment failed. Because of the refusal of further treatments, the patient died of rapid progression of ALL one month later. Finally, a new fusion transcript, *ACOT7–NPHP4*, was detected in the patient’s lymphoblastic leukemia cells via RNA sequencing.

**Conclusion:**

This is the first report of a patient with ALL carrying an *ACOT7–NPHP4* fusion gene. These findings may help understand the impact of *ACOT7–NPHP4* in clinical molecular monitoring and drug resistance to doxorubicin; furthermore, its leukemogenesis will be essential to explore in future.

**Supplementary Information:**

The online version contains supplementary material available at 10.1186/s12920-022-01378-7.

## Background

Acute lymphoblastic leukemia (ALL) is a subtype of hematological malignancies with an incidence of 1–1.5 per 100,000 people [1], which accounts for approximately 20% of adult ALL. In adult patients with ALL, the 5-year overall survival (OS) and event-free survival (EFS) are 30–40% and 30–45%, respectively [2, 3]. Currently, the complete remission (CR) rate in ALL is assessed mainly based on morphological criteria and immunophenotyping using flow cytometry [4]. Notably, molecular biomarkers such as *BCR–ABL* or *TCF3–PBX1* are more sensitive biomarkers for minimal residual disease (MRD) than morphology and immunophenotyping [5]. For instance, *BCR–ABL1* fusion-gene-guided diagnosis and targeted therapy with imatinib resulted in an improvement in 5-year disease-free survival (DFS) of 70% [6]; moreover, other fusion genes, including rearranged *KMT2A*, *ETV6–RUNX1*, *DUX4-IGH*, and *TCF3–PBX1* play important roles in the management of ALL. However, only 30–40% of patients with B- cell ALL and 10–20% of patients with T-cell ALL tested positive for chromosomal aberrations or chromosome translocations, which provided the basis for molecular marker-guided management [7].

With the development of next-generation sequencing (NGS), whole transcriptome sequencing (RNA-seq) has been widely used to identify the novel fusion genes [8]. At our center, patients with refractory/relapsed (R/R) ALL routinely undergo RNA-seq to identify new fusion genes as molecular biomarkers for monitoring and improving treatment. In the present case, one novel fusion gene, *ACOT7–NPHP4*, was detected in one patient with ALL. This fusion gene was subsequently characterized, and its chemoresistance was assessed.

## Case presentation

### Clinical course

A 34-year-old male patient was referred to the Department of Hematology, Second Hospital of Dalian Medical University in China on January 16, 2020. Clinically, he manifested fatigue and progressive painless lumps on both sides of the neck for over one month. Physical examination showed lymphadenomegaly on both sides of the neck, with sizes of 1.4 × 0.9 cm (right) and 1.5 × 1.1 cm (left); positive sternal tenderness; and no hepatomegaly or splenomegaly. Ultrasonic examination confirmed positive bilateral pleural effusion. Computed tomography (CT) found enlarged lymph nodes with a size of 43 × 21 mm located in the mediastinum, and a large variety of enlarged lymph nodes, the larger of which was 28 × 25 mm in size, spreading in the abdominal cavity, pelvic region, and retroperitoneum.

**Fig. 1:**
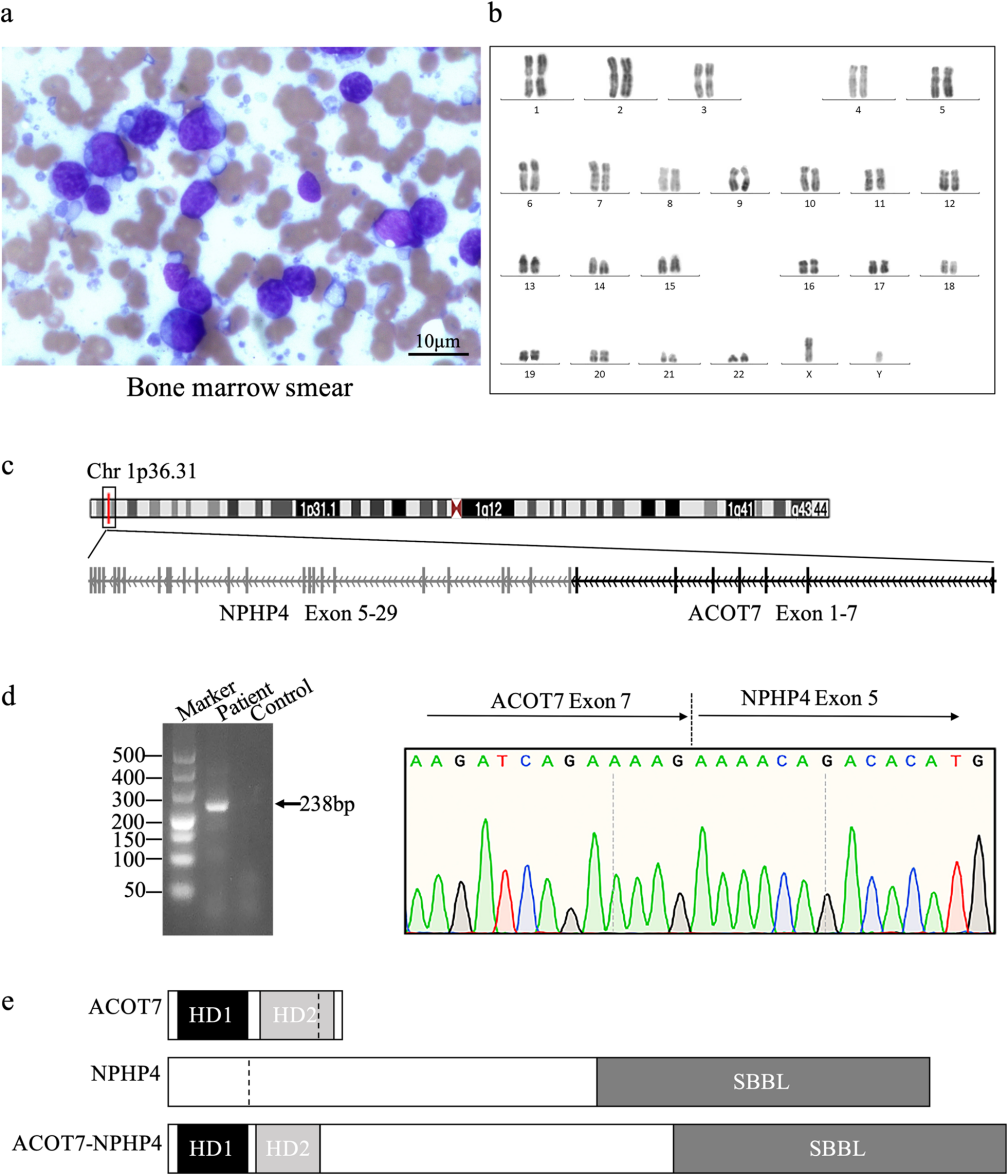
Identification of *ACOT7–NPHP4* in one patient with ALL. **a** May–Grunwald–Giemsa staining showed acute lymphoblastic leukemia blasts (87%) in a bone marrow (BM) smear with a magnification of 400. **b** Normal chromosomal karyotype with 46, XY. **c** The *ACOT7–NPHP4* transcript contained *ACOT7* exons 1–7 and *NPHP4* exons 5–29. **d** PCR band (238 bp) and the fusion site by Sanger sequencing of *ACOT7–NPHP4*. Control: patient’s hair follicle sample. **e** The domains of the wild-type *ACOT7* and *NPHP4*, and the domains of the *ACOT7–NPHP4* fusion proteins were shown. Breakpoints were indicated by the black dotted lines. HD1: HotDog *ACOT*-type 1; HD2: HotDog *ACOT*-type 2; SBBL: a domain named sufficient for basal bodies localization

A routine blood examination revealed white blood cell count of 6.5 × 10^9^/L, red blood cell count of 3.8 × 10^12^/L, hemoglobin (HB) level of 116.0 g/L, and platelet level of 188.0 × 10^9^/L. A bone marrow aspirate presented hypercellularity, with 87.0% lymphoblastic leukemia cells (Fig. 1a), and with normal karyotyping (46, XY) (Fig. 1b). Immunophenotyping showed lymphoblasts expressing CD34^+^, CD38^+^, CD19^+^, cCD79a+, CD7^+^, CD117^+^, HLA-DR^+^, and TdT^–^. NGS procedures as follows: mononucleated cells from marrow aspirate were collected for DNA extraction. A paired-end DNA sequencing library was prepared through gDNA shearing, end-repair, A-tailing, paired-end adaptor ligation, and amplification with a sequencing depth of 1000. NGS was performed on an Illumina HiSeq 2500 sequencing platform. NGS results showed that mononucleated cells harbored mutations in *ASXL1* (17.1%; NM_015338.6: exon12: c.1934dupG: p. G642fs rs1085307856), *PTPN11* (18.9%; NM_002834: exon3: c. A182T: p.D61V rs121918461), *RUNX1* (21.3%; NM_001754: exon9:c.C1274T: p. P425L), and *U2AF1* (15%; NM_006758: exon2:c.C101T: p.S34F rs371769427) genes. Based on clinical manifestations and laboratory examinations, the patient was newly diagnosed as common B- cell ALL.

Hyper-CVAD-L regimen, which has been described elsewhere, has a CR rate over 81% in induction therapy [9], was administered to this patient. The drug combination as follows: cyclophosphamide (300 mg/m^2^) was administered twice per day on days 2, 3, and 4; intravenous injection of vincristine (1.4 mg/m^2^) daily on days 1 and 11; pirarubicin (50 mg/m^2^) intravenously on day 4; dexamethasone (40 mg) daily on days 1 to 4 and days 11 to 14; and peg-asparaginase (3750 IU) intravenously on day 14. After two cycles of hyper-CVAD-L, 19.5% lymphoblastic leukemia cells remained in bone marrow, which carried the same mutations in *ASXL1* (11.5%; NM_015338.6: exon12: c.1934dupG: p. Gly646fs rs1085307856), *PTPN11* (10.38%; NM_002834: exon3: c.182 A > T: p. Asp61Val rs121918461), *RUNX1* (13.4%; NM_001754: exon9: c.1274 C > T: p. Pro425Leu), and *U2AF1* (12.86%; NM_006758: exon2: c.101 C > T: p. Ser34Phe rs371769427) genes, indicating a failure to induction therapy. Because the patient rejected further treatments, he then died of rapid progression of ALL one month later.

### **Identification of*ACOT7–NPHP4***

RNA sequencing with paired-end 150-bp read length was performed on the HiSeq 2500 platform with the sequencing depth over 100 M per sample. The sequencing data were mapped to the reference hg38 genome, and gene-level expression abundance was obtained using the Cufflinks package [10]. Furthermore, we detected the fusion transcripts among the RNA-seq data using STAR-Fusion. Finally, a new fusion transcript, *ACOT7–NPHP4*, was predicted in this patient.

The predicted breakage sites were located in exon 7 of the *ACOT7* gene and exon 5 of the *NPHP4* gene, respectively. The 5′ side of *ACOT7* joined into one part of 3′ side of *NPHP4*, yielding fusion transcript *ACOT7–NPHP4*, then it was confirmed by Sanger sequencing using polymerase chain reaction (PCR) products amplified with sense primer (F) (5′–CCAGTCCAGCTTGATC–3′) and antisense primer (R) (5′–TGGCTTCAGCGTGT–3′). Most of *ACOT7* domains and *NPHP4* domains remained in the fusion protein, as assessed based on an amino acid sequence analysis, suggesting that the novel fusion protein may have some aberrant function in lymphoblastic cells (Fig. 1c–e).

### **Drug resistance and monitoring of*ACOT7–NPHP4***

**Fig. 2:**
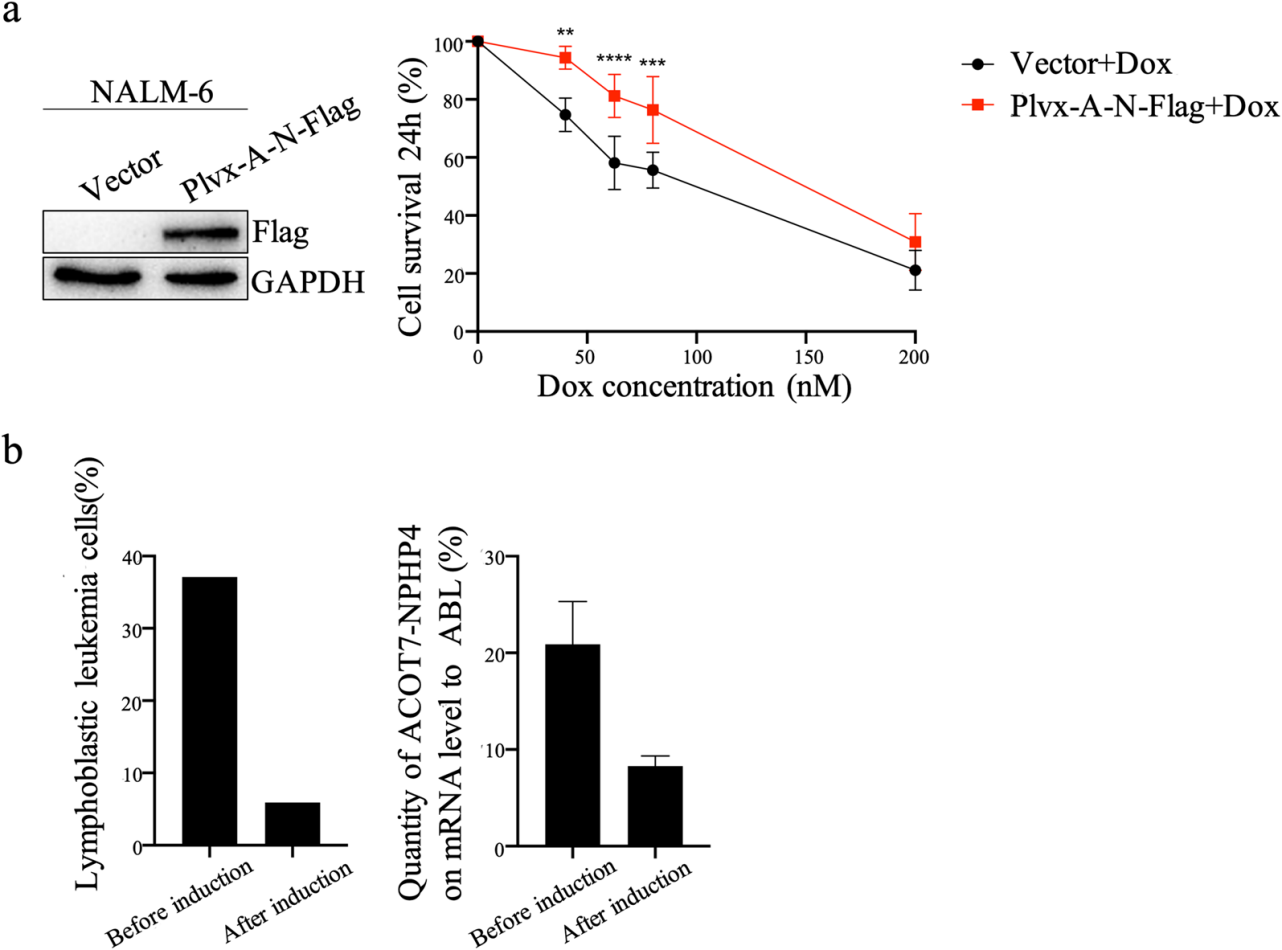
Survival of NALM-6 cells and expression of the *ACOT7–NPHP4*. **a** The transfection of the plvx-*ACOT7-NPHP4*-Flag (plvx-A-N-Flag) or vector plasmids into NALM-6 cells showed that NALM-6 cells carrying *ACOT7–NPHP4* (red line) presented a significant chemoresistance to doxorubicin after a 24-h exposure compared with NALM-6 cells carrying the vector plasmid (black line), as assessed using the cell counting kit-8 assay (mean ± SEM is displayed). **P < 0.01; ***P < 0.001; ****P < 0.0001. The expression of plvx-A-N-Flag in NALM-6 cells was tested via western blotting using an anti-Flag antibody. **b** The primary lymphoblastic leukemia cells from the patient were decreased after induction chemotherapy, as analyzed by flow cytometry in the bar chart on the left. The transcripts of *ACOT7–NPHP4* were decreased after induction therapy, as assessed by quantitative real-time PCR in the bar chart on the right

To investigate the chemoresistance to doxorubicin induced by *ACOT7–NPHP4*, NALM-6 cells (B-cell ALL cell line) were transiently transfected with plvx-*ACOT7-NPHP4*-Flag plasmid or the vector. The overexpression of *ACOT7–NPHP4* was verified by western blotting using an antibody against Flag (Fig. 2a). The cell survival was inhibited by doxorubicin in *ACOT7–NPHP4*-expressing cells, with a half maximal inhibitory concentration (IC50) of 82.5 nM, as assessed using the cell counting kit-8 assay; in contrast, the IC50 of doxorubicin in control cells was 69.96 nM, indicating that *ACOT7–NPHP4* led to a chemoresistance to doxorubicin. In addition, we also conducted drug resistance on other four substances of hyper-CVAD-L regimen. The results showed that there was no significant difference between *ACOT7–NPHP4*-expressing cells and control cells in cell viability inhibited by cyclophosphamide, vincristine, dexamethasone, or peg-asparaginase (Additional file 1: Fig. S1, Additional file 2: Fig. S2).

Furthermore, transcripts of *ACOT7–NPHP4* were detected by quantitative real-time PCR before and after the induction therapy in the patient. The aberrant expression decreased along with the reduction of ALL blast cells, as assessed by flow cytometry after induction therapy (Fig. 2b), indicating that *ACOT7–NPHP4* may serve as a molecular biomarker, and may have contributed to the failure to the induction therapy because of chemoresistance to doxorubicin.

## Discussion and conclusions

Fusion genes such as *BCR–ABL*, *DUX4-IGH*, or *TCF3–PBX1*, play critical roles in the diagnosis, targeted therapy, and prediction of prognosis in patients with leukemia. They also serve as molecular biomarkers and guide management in patients with ALL; for example, one retrospective investigation reported that 432 adult patients with Philadelphia chromosome-positive ALL in CR1 received allogeneic peripheral stem cell transplantation, among whom patients with detectable MRD of *BCR–ABL* before transplantation achieved 4-year OS of 55% and DFS of 46%, in contrast, patients with undetectable MRD of *BCR–ABL* reached OS of 67% and DFS of 60%, indicating that the molecular marker *BCR–ABL* was helpful to achieve better outcomes [11]. The patient belonged to high-risk group based on World Health Organization (WHO) classification and guidelines of National Comprehensive Cancer Network (NCCN) for ALL [5, 12], he had markedly systemic lymph node infiltration with no chromosomal abnormalities, however, conventional chemotherapy hyper-CVAD was ineffective. At our center, 26 patients with R/R ALL were performed with RNA-seq to identify new fusion genes as molecular biomarkers, therefore, a novel fusion gene, *ACOT7–NPHP4*, was predicted through RNA-seq and subsequently was confirmed.

*ACOT7–NPHP4* fusion gene detected in this patient comprised exons 1–7 of the *ACOT7* gene and exons 5–29 of the *NPHP4* gene. The HotDog folding structure presents in *ACOT7*, which is one of the main members of the *ACOT* family, is responsible for catalyzing the fatty acyl-CoA to free fatty acids and CoA-SH [13]. As reported elsewhere in 2019, high expression of wild-type (WT) *ACOT7* in 156 patients with acute myelogenous leukemia often yielded a poor prognosis, even after treatment with allogeneic peripheral stem cell transplantation [14]. However, little is known about the correlation between *ACOT7* and ALL, and our research reported that the breakage form of *ACOT7* gene was fused with *NPHP4* gene, with retaining functional domains. In addition, *NPHP4* gene is closely associated with nephronophthisis type 4, Senior–Loken syndrome type 4 [15], and tumorigenesis. To clarify the function of *ACOT7–NPHP4*, NALM-6 cells transfected with transcript *ACOT7–NPHP4* presented significant chemoresistance to doxorubicin, indicating that the new fusion gene may contribute to the failure to the induction therapy, to some extent. Moreover, *ACOT7–NPHP4* transcripts may serve as a new molecular biomarker to monitor the MRD in ALL patients. Notably, mutations in multiple genes, such as *ASXL1*, *PTPN11*, *RUNX1*, or *U2AF1*, have been described by Klaus H. Metzeler et al. to be risk factors for resistance to chemotherapy in leukemia, and these mutated genes are usually persistent among patients exhibiting treatment failure [16]. In the present case, these mutated genes, including *ASXL1*, *PTPN11*, *RUNX1*, and *U2AF1*, but particularly *ASXL1*, were persistent, as detected by NGS, after induction therapy, suggesting that *ASXL1* may partially contribute to chemoresistance, resulting in persistent *ASXL1* expression. Furthermore, the combination of the *ACOT7–NPHP4* fusion gene with *ASXL1* may have enhanced the chemoresistance, thus may in part lead to treatment failure in this patient with ALL.

Collectively, our results showed that a novel fusion gene, *ACOT7–NPHP4*, was identified, which potentially served as a molecular biomarker and enhanced chemoresistance to doxorubicin. Furthermore, the leukemogenesis of *ACOT7–NPHP4* in ALL should be further elucidated.

## Supplementary Information

**Additional file 1**: ** Fig. S1** Survival of NALM-6 cells inhibited by cyclophosphamide, vincristine, dexamethasone, or peg-asparaginase. The cell counting kit-8 assay showed that there was no significant difference in cell viability of 24-h inhibited by cyclophosphamide (CTX), vincristine (VCR), dexamethasone (Dex), or peg-asparaginase (Peg-ASNase) between plvx-A-N-Flag (red line) and vector plasmids (black line) into NALM-6 cells.

**Additional file 2**: ** Fig. S2** The original image of bone marrow smear. The original bone marrow smear was the field of view with a magnification of 100, and the red box was the field corresponding to Fig. 1a. The instrument of microscope was Olympus BX43, and the camera was Digital Camera: Smart V1050D. The image was taken at 300dpi resolution.

## Data Availability

The RNA-seq raw data has been deposited in the Gene Expression Omnibus (GEO) under GEO accession number GSE214117.

## Abbreviations

ALL: Acute lymphoblastic leukemia
CR: Complete remission
*ACOT7–NPHP4*: Acyl-CoA Thioesterase 7–Nephrocystin 4
RNA-seq: RNA sequencing
OS: Overall survival
EFS: Event-free survival
MRD: Minimal residual disease
DFS: Disease-free survival
NGS: Next-generation sequencing
R/R: Refractory/relapsed
CT: Computed tomography
HB: Hemoglobin
IC50: Half maximal inhibitory concentration
WHO: The World Health Organization
NCCN: The National Comprehensive Cancer Network
PCR: Polymerase chain reaction
WT: Wild-type
AML: Acute myelogenous leukemia
BM: Bone marrow
HD1: HotDog *ACOT*-type 1
HD2: HotDog *ACOT*-type 2
SBBL: A domain named sufficient for basal bodies localization
plvx-A-N-Flag: Plvx*-ACOT7-NPHP4*-Flag
CTX: Cyclophosphamide
VCR: Vincristine
Dex: Dexamethasone
Peg-ASNase: Peg-asparaginase

## References

[1] Jemal A, Tiwari RC, Murray T, Ghafoor A, Samuels A, Ward E, Feuer EJ, Thun MJ, American Cancer S (2004). Cancer statistics, 2004. CA Cancer J Clin.

[2] Paul S, Kantarjian H, Jabbour EJ (2016). Adult acute lymphoblastic leukemia. Mayo Clin Proc.

[3] Stock W, Johnson JL, Stone RM, Kolitz JE, Powell BL, Wetzler M, Westervelt P, Marcucci G, DeAngelo DJ, Vardiman JW (2013). Dose intensification of daunorubicin and cytarabine during treatment of adult acute lymphoblastic leukemia: results of Cancer and Leukemia Group B Study 19802. Cancer.

[4] Aldoss I, Stein AS (2018). Advances in adult acute lymphoblastic leukemia therapy. Leuk Lymphoma.

[5] Arber DA, Orazi A, Hasserjian R, Thiele J, Borowitz MJ, Le Beau MM, Bloomfield CD, Cazzola M, Vardiman JW (2016). The 2016 revision to the World Health Organization classification of myeloid neoplasms and acute leukemia. Blood.

[6] Alexander S (2014). Clinically defining and managing high-risk pediatric patients with acute lymphoblastic leukemia. Hematol Am Soc Hematol Educ Program.

[7] Bruggemann M, Kotrova M (2017). Minimal residual disease in adult ALL: technical aspects and implications for correct clinical interpretation. Hematol Am Soc Hematol Educ Program.

[8] Costa V, Aprile M, Esposito R, Ciccodicola A (2013). RNA-Seq and human complex diseases: recent accomplishments and future perspectives. Eur J Hum Genet.

[9] Garcia-Manero G, Kantarjian HM (2000). The hyper-CVAD regimen in adult acute lymphocytic leukemia. Hematol Oncol Clin N Am.

[10] Trapnell C, Williams BA, Pertea G, Mortazavi A, Kwan G, van Baren MJ, Salzberg SL, Wold BJ, Pachter L (2010). Transcript assembly and quantification by RNA-Seq reveals unannotated transcripts and isoform switching during cell differentiation. Nat Biotechnol.

[11] Nishiwaki S, Imai K, Mizuta S, Kanamori H, Ohashi K, Fukuda T, Onishi Y, Takahashi S, Uchida N, Eto T (2016). Impact of MRD and TKI on allogeneic hematopoietic cell transplantation for Ph + ALL: a study from the adult ALL WG of the JSHCT. Bone Marrow Transplant.

[12] Brown PA, Shah B, Advani A, Aoun P, Boyer MW, Burke PW, DeAngelo DJ, Dinner S, Fathi AT, Gauthier J (2021). Acute lymphoblastic leukemia, version 2.2021, NCCN clinical practice guidelines in oncology. J Natl Compr Cancer Netw.

[13] Jung SH, Lee HC, Hwang HJ, Park HA, Moon YA, Kim BC, Lee HM, Kim KP, Kim YN, Lee BL (2017). Acyl-CoA thioesterase 7 is involved in cell cycle progression via regulation of PKCzeta–p53–p21 signaling pathway. Cell Death Dis.

[14] Zhang X, Liu B, Zhang J, Yang X, Zhang G, Yang S, Wang J, Shi J, Hu K, Wang J, Jing H (2019). Expression level of ACOT7 influences the prognosis in acute myeloid leukemia patients. Cancer Biomark..

[15] Otto E, Hoefele J, Ruf R, Mueller AM, Hiller KS, Wolf MT, Schuermann MJ, Becker A, Birkenhager R, Sudbrak R, Hennies HC (2002). A gene mutated in nephronophthisis and retinitis pigmentosa encodes a novel protein, nephroretinin, conserved in evolution. Am J Hum Genet..

[16] Rothenberg-Thurley M, Amler S, Goerlich D, Kohnke T, Konstandin NP, Schneider S, Sauerland MC, Herold T, Hubmann M, Ksienzyk B, Zellmeier E (2018). Persistence of pre-leukemic clones during first remission and risk of relapse in acute myeloid leukemia. Leukemia..

