# Supplementary figures and images for: One novel ACOT7–NPHP4 fusion gene identified in one patient with acute lymphoblastic leukemia: a case report
Source: BMC Med Genomics. 2022 Oct 31;15:226. doi: 10.1186/s12920-022-01378-7 (PMC9620589; doi:10.1186/s12920-022-01378-7)

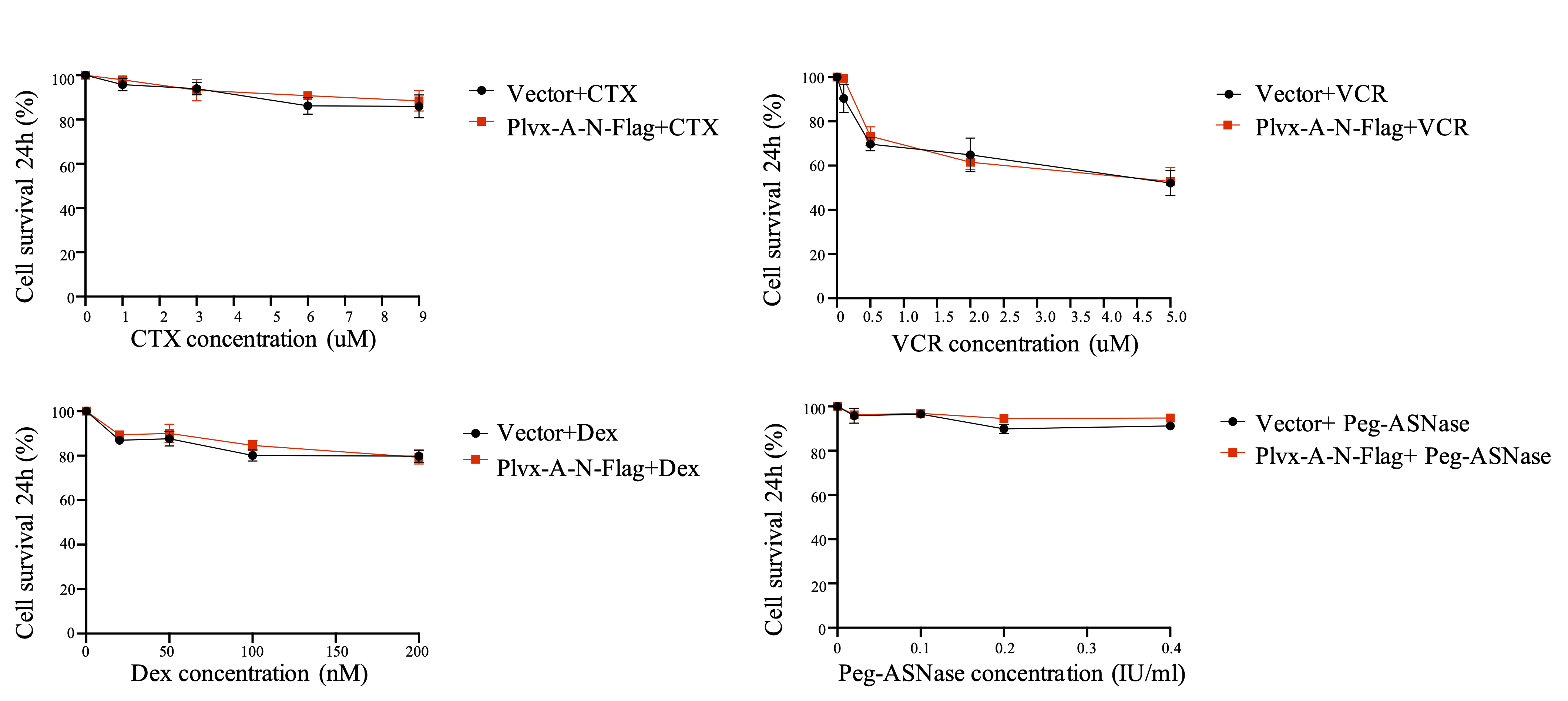

Supplement: Supplementary file 1 — Additional file 1: Fig. S1 Survival of NALM-6 cells inhibited by cyclophosphamide, vincristine, dexamethasone, or peg-asparaginase. The cell counting kit-8 assay showed that there was no significant difference in cell viability of 24-h inhibited by cyclophosphamide (CTX), vincristine (VCR), dexamethasone (Dex), or peg-asparaginase (Peg-ASNase) between plvx-A-N-Flag (red line) and vector plasmids (black line) into NALM-6 cells. [file 12920_2022_1378_MOESM1_ESM.tiff]

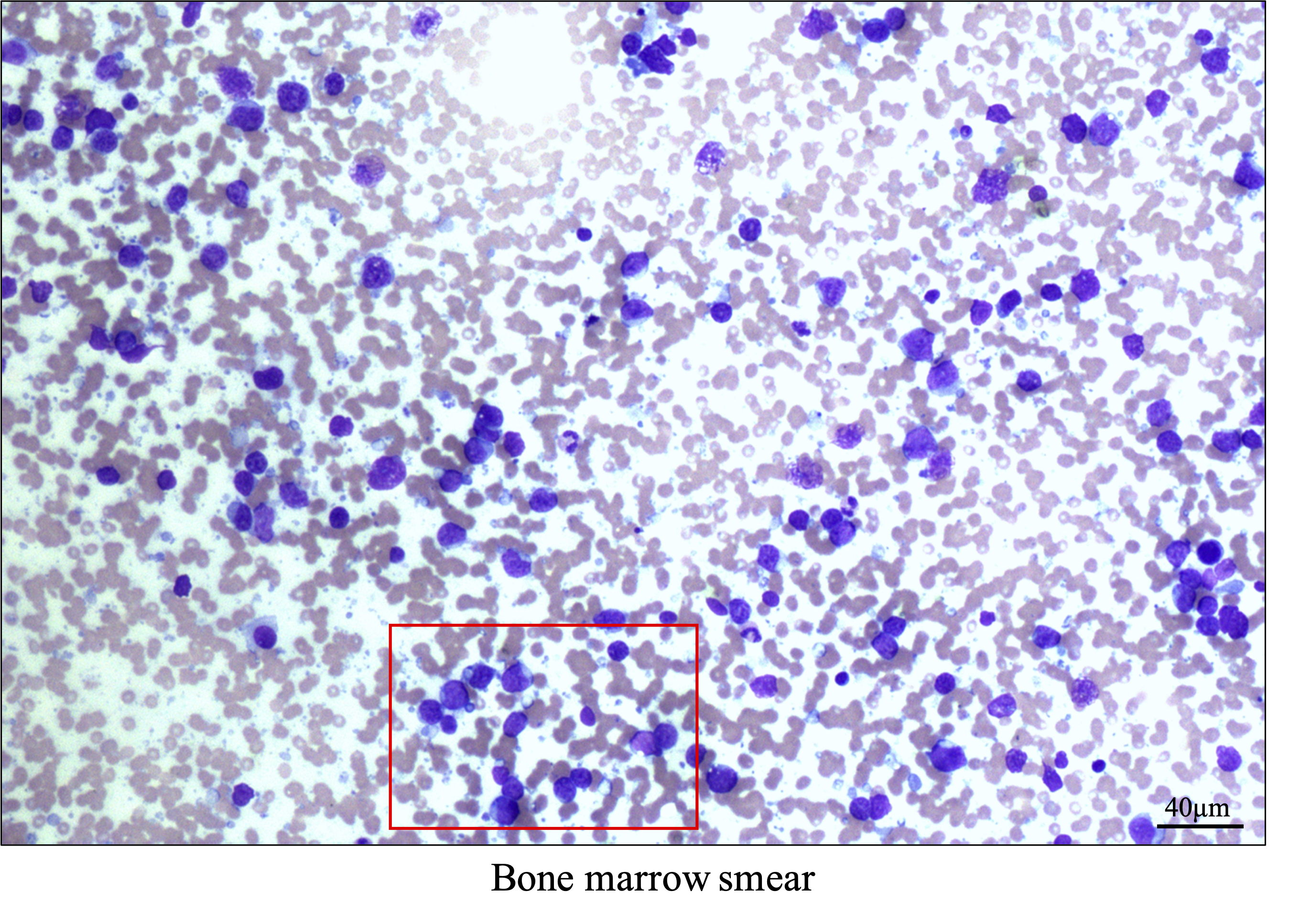

Supplement: Supplementary file 2 — Additional file 2: Fig. S2 The original image of bone marrow smear. The original bone marrow smear was the field of view with a magnification of 100, and the red box was the field corresponding to Fig. 1a. The instrument of microscope was Olympus BX43, and the camera was Digital Camera: Smart V1050D. The image was taken at 300dpi resolution. [file 12920_2022_1378_MOESM2_ESM.tiff]
